# Increased leukotriene B4 plasma concentration in type 2 diabetes individuals with cardiovascular autonomic neuropathy

**DOI:** 10.1186/s13098-020-00606-3

**Published:** 2020-11-12

**Authors:** Jose Antonio Januario Neves, Mozânia Reis De Matos, Theresa Ramalho, Daniele Pereira Santos-Bezerra, Cristiane Das Graças Dias Cavalcante, Renata D’ Alpino Peixoto, Márcia Silva Queiroz, Sonia Jancar, Maria Lucia Correa-Giannella

**Affiliations:** 1 Programa de Pos-Graduação Em Medicina, Universidade Nove de Julho (UNINOVE), Rua Vergueiro 235, 2° subsolo, Pos-graduação, Sao Paulo, CEP: 01504-001 Brazil; 2 Unidade Básica de Saúde Dra. Ilza Weltman Hutzler. Rua Coronel Walfrido de Carvalho, Sao Paulo, CEP: 02472-180 Brazil; 3 Laboratório de Imunofarmacologia, Departamento de Imunologia, Instituto de Ciências Biomédicas, Universidade de São Paulo, Av. Prof. Lineu Prestes, 1730, Sao Paulo, CEP: 05508-900 Brazil; 4 Laboratório de Carboidratos E Radioimunoensaio (LIM-18) Do Hospital das Clínicas HCFMUSP, Faculdade de Medicina, Universidade de São Paulo, Avenida Dr. Arnaldo, 455, Sala 3321, Sao Paulo, CEP: 01246-903 Brazil

**Keywords:** LTB4, Chronic diabetes complications, Inflammation, Insulin resistance

## Abstract

**Background and aim:**

A low-grade inflammation is associated with cardiac autonomic neuropathy (CAN) and increased concentration of leukotriene B4 (LTB4) was found in individuals with type 1 diabetes and definitive CAN. This cross-sectional study evaluated plasma concentration of LTB4 and of other inflammatory mediators, namely, tumor necrosis factor (TNF), interleukin (IL)1B, and IL10 in individuals with type 2 diabetes (T2D) and different degrees of CAN, and correlated these inflammatory mediators with the degree of glycemic control and with a surrogate marker of insulin resistance.

**Methods:**

TNF, IL1B, IL10 and LTB4 plasma concentrations were measured in 129 T2D subjects (62% women with [median] age of 63 years, disease duration of 8 years and HbA1c of 7.3%) with or without CAN. The Lipid accumulation product index was used as a surrogate marker of insulin resistance.

**Results:**

LTB4 concentration was significantly higher in those presenting incipient CAN (69.7 ± 16.6 pg mL^-1^) and definitive CAN (71.5 ± 15.7 pg mL^-1^) *versus* those without CAN (57.0 ± 13.9 pg mL^-1^). The groups without CAN and with incipient CAN were pooled (group without definitive CAN) and compared to those with definitive CAN. LTB4 concentration was higher in the latter group, as well as TNF concentration, while IL10 concentration was lower in this group. After adjustment for confounding variables, only LTB4 concentration remained significantly different between the groups with and without definitive CAN. Plasma concentration of LTB4 did not correlate with the degree of glycemic control. After sorting the participants by sex, a borderline weak correlation was found between LTB4 and the Lipid accumulation product index in women.

**Conclusion:**

In the T2D setting, circulating LTB4 concentration seems to be associated with cardiovascular dysautonomia.

## Background

Chronic low-grade inflammation is present in obesity, being closely associated with the etiopathogenesis of insulin resistance and, consequently, with type 2 diabetes mellitus (T2D). Among the pro-inflammatory cytokines produced by the adipose tissue in visceral obesity are tumor necrosis factor (TNF), interleukin (IL) 1B and IL6. On the other hand, in eutrophic individuals, the adipose tissue secretes anti-inflammatory cytokines, such as IL10 [1]. Studies in rodents have also implicated leukotriene B4 (LTB4) in the insulin resistance triggered by obesity [2].

Inflammation is also implicated in the etiopathogenesis of chronic complications of diabetes mellitus (DM). TNF and IL6 are produced by endothelial, mesangial and leukocyte cells, having already been associated with the development and progression of diabetic kidney disease and diabetic macroangiopathy [3].

Recently, we reported that in individuals with type 1 DM (T1D), those with worse glycemic control had higher plasma concentration of LTB4. Additionally, the analyses of this inflammatory marker according to the status of microvascular complications showed a higher plasma concentration of LTB4 in individuals with definitive cardiovascular autonomic neuropathy (CAN) as compared to individuals without this complication [4]. This finding possibly reflects a lower parasympathetic activity secondary to CAN, with impairment of the inflammatory reflex, a physiological mechanism by which the vagus nerve regulates the immune function and inhibits the excessive production of pro-inflammatory mediators [5].

In the present study, we aimed to evaluate the plasma concentrations of LTB4 and other inflammatory mediators, namely, TNF, IL1B and IL10 in individuals with T2D with and without CAN. In addition, we correlated the concentrations of these inflammatory mediators with the degree of glycemic control and with a surrogate marker of insulin resistance.

## Methods

In this cross-sectional study, 129 individuals with T2D were selected from a cohort of 551 individuals recruited from a primary care unit in the city of São Paulo and evaluated for the CAN status between September 2018 and February 2019, as previously described. Briefly, a total of 583 out of the 1,853 T2D individuals followed in the unit agreed to participate; 32 individuals were not included because their capillary blood glucose was > 180 mg/dL^−1^ at the day of evaluation (hyperglycemia may interfere with the results of the autonomic tests) [6]. These 129 individuals were selected in order to form three groups matched for sex, age, DM duration and HbA1c: without CAN (n = 44), with incipient CAN (n = 41) and with definitive CAN (n = 44). Thus, this study was performed in a convenience sample, since there was no sample size calculation. The groups without CAN and with incipient CAN were pooled (group without definitive CAN) and compared to those with definitive CAN. Participants were evaluated for demographic, clinical and biochemical characteristics, and for CAN status (by Ewing tests combined with spectral analysis of the heart rate [HR]). The diagnosis of incipient and definitive CAN was made, respectively, in presence of 2 and of ⩾3 abnormalities of HR variability and Ewing tests, as previously described [7]. The Lipid accumulation product (LAP) index was calculated as a surrogate marker of insulin resistance: (waist circumference [WC, in cm]–65) x triglycerides [TG, in mmol L^−1^] in men and (WC–58) x TG in women [8, 9]. Plasma concentrations of TNF, IL1B, and IL10 were measured by BD Opt EIA ELISA Kit II (BD Biosciences, CA, USA) and LTB4 concentration was measured by the EIA kit (Cayman Chemical, MI, USA), according to the manufacturer’s instructions. The statistical analyses were performed with JMP software version 8.0 (SAS Institute, Cary, NC, USA). The results are expressed as median ± interquartile interval, except for the plasma concentrations of the inflammatory mediators, which are expressed as mean ± standard deviation (SD). The differences between the groups with and without definitive CAN were assessed by Pearson’s χ^2^ for the categorical variables and by Wilcoxon’s test for the continuous variables. The Wilcoxon Kruskal–Wallis test followed by Dunn's post-test was employed to identify differences in the concentrations of the inflammatory mediators between the groups without CAN, with incipient and with definitive CAN. Logistic regression analyses with adjustment for confounding variables were employed to evaluate plasma concentrations of the inflammatory mediators between the groups with and without definitive CAN. Correlation analyses were performed by the Spearman’s rank correlation coefficient. A *P* value of < 0.05 was considered statistically significant.

## Results

**Fig. 1:**
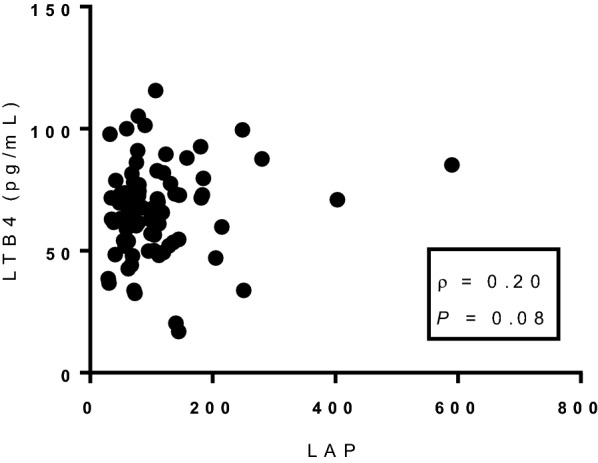
Correlation between plasma leukotriene B4 (LTB4) concentrations and the Lipid accumulation product (LAP) index in women with type 2 diabetes mellitus

The characteristics of T2D individuals according to CAN status is shown in Table 1. TG concentration and the LAP index presented borderline differences between those with and without definitive CAN (Fig. 1).

**Table 1:** Demographic, clinical and biochemical characteristics of type 2 diabetes individuals sorted according to the status of cardiovascular autonomic neuropathy (CAN)

Demographic. clinical and biochemical characteristics	Without definitive CAN	With definitive CAN	*P* value
*n*	*85*	*44*	
Age (years)	63 (60–67)	62 (58–67)	0.43
Sex (% female)	60	65	0.56
Ethnicity (Caucasoid/Negroid/Asiatic) (%)	68/28/4	73/27/0	0.60
Body mass index (kg m^2^)	29.6 (26.4–35.7)	28.8 (25.9–33.3)	0.35
Waist circumference (cm)	103 (97–115)	102 (96–110)	0.91
Arterial hypertension (%)	74	84	0.19
Smoking (%)	10.6	13.6	0.61
Total cholesterol (mg dL^−1^)	193 (165–234)	203 (167–233)	0.60
HDL (mg dL^−1^)	47 (38–56)	44 (35–52)	0.16
LDL (mg dL^−1^)	118 (94–150)	113 (89–147)	0.52
Triglycerides (mg dL^−1^)	*144 (111–218)*	*184 (132–212)*	*0.05*
Lipid accumulation product index	*71.1 (54.4–110.4)*	*80.5 (60.1–151.4)*	*0.06*
*Diabetes status*			
Diabetes duration (years)	8 (4–13)	9 (4–20)	0.34
HbA_1C_ (%)	7.3 (6.2–9.2)	7.5 (6.4–9.1)	0.94
(mmol.mol^−1^)	56 (44–77)	58 (46–76)	
eGFR < 60 mL.min^−1^.1.73m^2^ (%)	16	11	0.57
Distal symmetric polyneuropathy (%)	11.7	13.6	0.75
*Inflammatory mediators*			
Leukotriene B4 (pg mL^−1^)	*63.1* ± *16.4*	*71.5* ± *15.7*	*0.006*
Tumor necrosis factor (pg mL^−1^)	*1.9* ± *7.9*	*7.4* ± *36.0*	*0.001*
Interleukin 1B (pg mL^−1^)	23.6 ± 106.3	9.2 ± 18.1	0.32
Interleukin 10 (pg mL^−1^)	*5.5* ± *13.5*	*3.8* ± *14.2*	*0.002*

Data are expressed as median (interquartile range), except for the inflammatory mediators, which are expressed as mean ± SD. eGFR: estimated glomerular filtration rate

HDL: High-density lipoprotein; LDL: Low-density lipoprotein. Arterial hypertension defined as systolic/diastolic blood pressure ≥ 140/90 mmHg or use of anti-hypertensive drugs not for renal protection purposes; Hypercholesterolemia defined as an LDL > 100 mg.dL^−1^ or use of statin. Differences between groups were assessed by the Wilcoxon and by the Pearson’s χ^2^tests. Significantly different variables are shown in italic

Plasma concentrations of TNF, IL10 and LTB4 differed significantly among the groups without CAN, with incipient CAN and with definitive CAN. For TNF and IL10, Dunn's post-test did not show differences between groups while concentration of LTB4 was higher in those presenting incipient CAN (69.7 ± 16.6 pg mL^−1^) and definitive CAN (71.5 ± 15.7 pg mL^−1^) *versus* those without CAN (57.0 ± 13.9 pg mL^−1^). When the groups with and without definitive CAN were compared, TNF and LTB4 concentrations were significantly higher in the group with definitive CAN while IL10 concentration was significantly lower in this group in comparison to those without definitive CAN (Table 1). After adjustment for confounding variables, only LTB4 concentration remained significantly different between the groups with and without definitive CAN (Table 2).

**Table 2:** Association between inflammatory mediators and definitive cardiac autonomic neuropathy after adjustment for confounding variables

		TNF	IL10	IL1B	LTB4
Model 1	Unadjusted	*P* = *0.001*	*P* = *0.002*	*P* = 0.32	*P* = *0.006*
Model 2	Adjusted for sex and age	*P* = 0.18	*P* = 0.47	*P* = 0.51	*P* = *0.006*
Model 3	Adjusted for sex, age and body mass index (BMI)	*P* = 0.83	*P* = 0.32	*P* = 0.60	*P* = *0.001*
Model 4	Adjusted for sex, age, BMI, and waist circumference (WC)	*P* = 0.17	*P* = 0.54	*P* = 0.93	*P* = *0.016*
Model 5	Adjusted for sex, age, BMI, WC, diabetes duration, HbA1c, cholesterol, triglycerides, arterial hypertension, eGFR, and use of ACEi	*P* = 0.67	*P* = 0.29	*P* = 0.30	*P* = *0.013*

ACEi: Angiotensin-converting enzyme inhibitors; eGFR: estimated glomerular filtration rate; IL: Interleukin, LTB4: Leukotriene B4; TNF: Tumor necrosis factor. Significantly different *P* values are shown in italic

Plasma concentration of LTB4 did not correlate with the degree of glycemic control as evaluated by HbA1c (⍴ = − 0.015; *P* = 0.867) or with insulin resistance as evaluated by the LAP index (⍴ = − 0.093; *P* = 0.2985). However, when T2D individuals were sorted by sex, a borderline weak correlation was found between LTB4 concentration and the LAP index in women (⍴ = 0.200; *P* = 0.082) (Fig. 1).

## Discussion

Individuals with T2D and definitive CAN presented higher LTB4 plasma concentration than those without this chronic complication. Differences in plasma concentrations of TNF and IL10 between groups with and without CAN lost statistical significance after adjustment for sex and age. On the other hand, LTB4 concentration remained significantly different between the two groups even after adjustment for confounding variables that may interfere with inflammatory markers, such as anthropometric (BMI and WC) and metabolic (glycemic control and dyslipidemia) factors. In the study by Herder et al. for instance, the high concentrations of C-reactive protein and IL6, that were associated with cardiovascular autonomic dysfunction in the unadjusted model, lost statistical significance after adjusting for anthropometric and metabolic variables, showing that the baseline conditions of individuals with T2D by themselves are already associated with subclinical inflammation [10]. In the present study, the increased LTB4 concentration can be attributed to CAN, since adjustment for confounding variables did not alter the association. These findings are in agreement with the ones reported in T1D individuals and corroborate the modulation of the leukotriene pathway by cardiovascular dysautonomia.

Differently from what was observed in T1D individuals, LTB4 plasma concentration did not correlate with glycemic control [4]. A weak borderline correlation was observed between LTB4 concentration and the LAP index in women. Although LTB4 has already been implicated in insulin resistance in rodent models [2, 11, 12], to the best of the authors' knowledge, there are no clinical studies correlating plasma LTB4 concentration with markers of insulin resistance. Thus, studies in larger independent populations are warranted to further investigate the participation of LTB4 in this metabolic derangement, since the present study has a small sample size as its main limitation.

## Conclusion

In the T2D setting, circulating LTB4 concentration seems to be associated with CAN. As already proposed for T1D [4], this inflammatory mediator may exacerbate the cardiovascular burden imposed by CAN, since there is evidence from preclinical studies that LTB4 participates in the development of atherosclerosis [13] by inducing chemoattraction of monocytes and their conversion to foam cells [14] and smooth muscle cells migration and proliferation. The expression of LTB4 receptors in human atherosclerotic lesions [15] and the inhibition of lesions progression by an antagonist of LTB4 receptors in a mouse atherosclerosis model [16] corroborate the pro-atherogenic effects of this lipid mediator.

## Data Availability

The data used to support the findings of this study are available from the corresponding author upon request.

## Abbreviations

ACEi: Angiotensin-converting enzyme inhibitors
BMI: Body mass index
CAN: Cardiovascular autonomic neuropathy
DM: Diabetes mellitus
eGFR: Estimated glomerular filtration rate
EIA: Enzyme immunoassays
ELISA: Enzyme-Linked Immunosorbent Assay
HDL: High-density lipoprotein
HR: Heart rate
IL: Interleukin
LAP: Lipid accumulation product
LDL: Low-density lipoprotein
LTB4: Leukotriene B4
SD: Standard deviation
T1D: Type 1 diabetes mellitus
T2D: Type 2 diabetes mellitus
TG: Triglycerides
TNF: Tumor necrosis fator
WC: Waist circumference
